# Effectiveness and safety of low‐dose apatinib in advanced gastric cancer: A real‐world study

**DOI:** 10.1002/cam4.3105

**Published:** 2020-05-22

**Authors:** Yingying Du, Qisheng Cao, Congqiao Jiang, Hui Liang, Zhongliang Ning, Chushu Ji, Jinguo Wang, Chaoping Zhou, Zonghui Jiang, Changjun Yu, Lei Li, Yong Zhao, Yuemei Xu, Tengyun Xu, Wenjun Hu, Daoqin Wang, Huaidong Cheng, Guihe Wang, Jinhua Zhou, Song Wang, Yanshun Zhang, Zhiqiang Hu, Xinzhong Li, Donghui Lu, Jun Zhang, Hua Xie, Guoping Sun

**Affiliations:** ^1^ Department of Oncology The First Affiliated Hospital of Anhui Medical University Hefei China; ^2^ Department of Interventional Oncology People's Hospital of Ma'anshan Ma'anshan China; ^3^ Department of Gastrointestinal Surgery The First Affiliated Hospital of Bengbu Medical College Bengbu China; ^4^ Department of Tumor Radiotherapy Lu'an Hospital of Traditional Chinese Medicine Lu'an China; ^5^ Department of Gastrointestinal Surgery The First Affiliated Hospital of University of Science and Technology of China (Anhui Provincial Cancer Hospital) Hefei China; ^6^ Department of Oncology The First Affiliated Hospital of University of Science and Technology of China Hefei China; ^7^ Department of Gastrointestinal Surgery Yijishan Hospital of WanNan Medical College Wuhu China; ^8^ Department of Surgical Oncology Anqing Municipal Hospital Anqing China; ^9^ Department of Oncology The First People's Hospital of Chuzhou City Chuzhou China; ^10^ Department of Gastrointestinal Surgery The First Affiliated Hospital of Anhui Medical University Hefei China; ^11^ Department of Surgical Oncology The First Affiliated Hospital of Bengbu Medical College Bengbu China; ^12^ Department of Oncology Lu'an Hospital of Traditional Chinese Medicine Lu'an China; ^13^ Department of Oncology Jinzhai Country Hospital of Traditional Chinese Medicine Lu'an China; ^14^ Department of Oncology People's Hospital of Fuyang City Fuyang China; ^15^ Department of Gastrointestinal Surgery Wanbei Coal‐Electricity Group General Hospital Suzhou China; ^16^ Department of Oncology The Second Affiliated Hospital of Anhui Medical University Hefei China; ^17^ Department of Gastrointestinal Surgery People's Hospital of Tongling City Tongling China; ^18^ Department of Oncology The First Affiliated Hospital of Anhui University of Chinese Medicine Hefei China; ^19^ Department of Oncology Huainan First People's Hospital Huainan China; ^20^ Department of Oncology Huaibei Miners General Hospital Huaibei China; ^21^ Department of Oncology The People's Hospital of Huaibei Huaibei China; ^22^ Department of Oncology The 901 Hospital of the Joint Logistic Support Force of the People's Liberation Army of China Hefei China; ^23^ Department of Oncology The Second People's Hospital of Wuhu Wuhu China; ^24^ Department of Oncology The People’s Hospital of Xuancheng City Xuancheng China

**Keywords:** advanced gastric cancer, apatinib, real‐world

## Abstract

Apatinib has been demonstrated to be effective and safe among patients with gastric cancer failing after at least two lines chemotherapy. This study aimed to evaluate its effectiveness and safety of low‐dose apatinib for the treatment of gastric cancer in real‐world practice. We performed a prospective, multicenter observation study in a real‐world setting. Patients with advanced gastric cancer more than 18 years old were eligible and received low‐dose apatinib (500 mg or 250mg per day) therapy. The median progression‐free survival (PFS), median overall survival (OS), objective response rate (ORR), disease control rate (DCR), and safety were assessed. Between September 2017 and April 2019, a total of 747 patients were enrolled. The mPFS was 5.56 months (95% CI 4.47‐6.28), and mOS was 7.5 months (95% CI 6.74‐8.88). Four patients achieved complete response, 47 achieved partial response, and 374 patients achieved stable disease. The ORR was 6.83% and DCR was 56.89%. In addition, multivariate Cox regression analysis indicated that hand‐foot syndrome was one independent predictor for PFS and OS. The most common adverse events (AEs) at any grade were hypertension (36.55%), proteinuria (10.26%), hand‐foot syndrome (33.53%), fatigue (24.9%), anemia (57.35%), leukopenia (44.49%), thrombocytopenia (34.21%), and neutropenia (53.33%). Grade 3‐4 AEs with incidences of 5% or greater were anemia (13.97%), thrombocytopenia (7.14%), and neutropenia (6.67%). No treatment‐related death was observed during the treatment of apatinib. The prospective study suggested that low‐dose apatinib was an effective regimen for the treatment of advanced gastric cancer with tolerable or controlled toxicity in real world.

Trial registration: NCT03333967.

## Graphical abstract

Apatinib has been demonstrated to be effective and safe among patients with gastric cancer failing after at least two lines chemotherapy. The present study aimed to evaluate its effectiveness and safety of low‐dose apatinib for the treatment of gastric cancer in real‐world practice. The prospective study suggested that low‐dose apatinib was an effective regimen for the treatment of advanced gastric cancer with tolerable or controlled toxicity in real world.

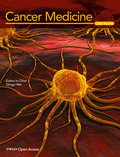

## 1 INTRODUCTION

Gastric cancer is one of the most common digestive system malignancy, with high morbidity and mortality worldwide, which is next only to lung cancer and liver cancer.^1^ For the early stage patients, surgery is the best treatment, however, approximately 80% of patients are initially diagnosed incurable due to locally advanced or metastatic gastric cancer.^2^ With the development of chemotherapeutic drugs, targeted drugs, and immunotherapy, the median survival of advanced gastric cancer has been extended to more than 12 months.^3^ However, the survival rate of 5 years remain poor with <10%.^2^ Thus, exploring novel therapy strategies are necessary.

Angiogenesis has been investigated widely and known as its contribution to initial and development of tumor.^4^ Vascular endothelial growth factor receptors (VEGFR), including VEGFR‐1, VEGFR‐2, VEGFR‐3, and VEGFR‐4, have been demonstrated to bind the vascular endothelial growth factor (VEGF) family to motivate angiogenesis signal pathway, and function important role as critical regulators of angiogenesis.^4^ Apatinib, as a specifically targeting VEGFR‐2 and oral receptor tyrosine kinase inhibitor, has been shown that it could inhibit the angiogenesis of tumor through prohibiting VEGF‐promoted tumor development. Also, Li et al have reported that apatinib expressively advanced overall survival (OS) and PFS with tolerable toxicities in chemotherapy‐refractory advanced gastric cancer after failing at least two lines chemotherapy in the randomized, double‐blind, placebo‐controlled phase III trial and a randomized, placebo‐controlled, parallel‐arm, phase II trial,^5^, ^6^ but the dose of 850 mg daily or 425 mg twice a day in the two trials resulted in many severe adverse events in clinical practice. Additionally, the dosage of 500 mg daily in clinical practice was commonly used mainly due to the concern of potential grade 3‐4 adverse events such as hypertension, proteinuria, and hand‐foot syndrome.

Preliminary data of this present have been released in ASCO 2019 (Abstract, 161)^7^ and ESMO 2018 Congress (683P).^8^ So, we further analyzed this prospective observation study in order to provide more clinical proof for the use of low‐dose apatinib in patients with advanced gastric cancer.

## 2 PATIENTS AND METHODS

### 2.1 Patients

Patients receiving apatinib were included in this study from 23 centers in China. All men or women older than 18 years of age who pathologically or histologically confirmed advanced or metastatic adenocarcinoma of stomach were included. Patients with pregnancy or lactation, those with contraindications or allergy for apatinib, and those unsuitable to this study were excluded. All patients provided written informed consent before participating in the study. This study was approved by the local ethics committee of all hospitals.

### 2.2 Study design and treatment

This was a prospective, multicenter observation study in a real‐world setting. All patients received apatinib therapy by an oral administration once a day and the dose (500 mg or 250 mg) could be adjusted according to patient's performance status or adverse event.

### 2.3 Efficacy and safety

Clinical responses were evaluated by computed tomography (CT) or magnetic resonance imaging (MRI) until disease progression. The responses were classified as complete response (CR), partial response (PR), stable disease (SD), and progressive disease (PD) according to Response Evaluation Criteria in Solid Tumors (RECIST) 1.1 criteria. The objective response rate (ORR) or disease control rate (DCR) was computed as the addition of CRs plus PRs or CRs plus PRs plus SDs, respectively. Survival status was followed up every 3 months to analyze the PFS and OS. The PFS or OS was defined as time from the start of apatinib administration until disease progression or death of any cause death according to RECIST 1.1, respectively. All treatment‐related adverse events (AEs) were defined and graded according to the National Cancer Institute Common Terminology Criteria for Adverse Events (version 3.0).

### 2.4 Statistical analyses

The Kaplan‐Meier method and log‐rank test were used to analyze the PFS and OS. Multivariate analyses were performed with the Cox's proportional hazards regression model to explore the potential factors for PFS and OS. All the statistical analyses were performed using SAS 9.2 software (SAS Institute).

## 3 RESULTS

### 3.1 Patient characteristics

Between September 1, 2017 and April 15, 2019, 747 patients with advanced gastric cancer who received the treatment of apatinib were included in the FAS population. The patients’ characteristics were shown in Table 1. All patients included 547 male and 200 female patients with the mean age of 62.33 years. In addition, 711 and 36 patients showed an ECOG performance status of 0/1 (95.18%) and ≥2 (4.82%), respectively. 58.63% of the included patients had metastases. The patients had experienced previous therapy such as gastrostomy (55.69%), chemotherapy (68.94%), and radiotherapy (2.41%). A total of 611 patients received the initial dose of 500 mg and 136 patients were treated with the initial dose of 250 mg.

**TABLE 1:** Baseline characteristics of patients

Characteristics	N (%)
Age (y)	
Mean ± SD	62.33 ± 11.10
Sex	
Male	547 (73.23)
Female	200 (26.77)
ECOG PS	
0	28 (3.75)
1	683 (91.43)
≥2	36 (4.82)
Clinical stage	
III	101 (13.52)
IV	489 (65.46)
Unknown	157 (21.02)
Differentiation	
Poorly	323 (43.24)
Moderately	125 (16.73)
Highly	5 (0.67)
Other	283 (37.88)
Initial dose	
500 mg	611 (81.8%)
250 mg	136 (18.2%)
Metastases	
Yes	438 (58.63)
No	309 (41.37)
Number of metastases	
≤2	331 (77.70)
>2	95 (22.30)
Previous anticancer treatment	
Surgery	416 (55.69)
Radiotherapy	18 (2.41)
Chemotherapy	515 (68.94)

Abbreviations: ECOG, Eastern Cooperative Oncology Group; PS, performance status.

### 3.2 Effectiveness

A total of 516 patients were assessed by an imaging examination (CT or MRI). Among them, 4 patients showed CR, 47 patients (6.29%) achieved PR, 374 patients (50.06%) showed stable disease, and 91 patients (12.18%) were evaluated as progression disease after the treatment of apatinib. These data exhibited an ORR of 6.83% and a DCR of 56.89%. The specifics of the clinical responses were listed in Table 2.

**TABLE 2:** Tumor responses

Response	N (n = 747)	Percentage (%)
CR	4	<1
PR	47	6.29
SD	374	50.06
PD	91	12.18
Not evaluable	231	30.92
ORR	51	6.83
DCR	425	56.89

Abbreviations: CR, complete response; DCR, disease control rate; ORR, objective response rate; PD, progression disease; PR, partial response; SD, stable disease.

For survival outcome, as shown in Table 3, the median PFS was 5.56 months (95% CI 4.74‐6.28), and the 6‐month and 12‐month PFS rate were 47.04% (95% CI 42.93‐51.05) and 22.85% (95% CI 18.40‐27.60), respectively. The median OS was 7.5 months (95% CI 6.74‐8.88), and the 6‐month and 12‐month OS rate were 58.54% (95% CI 54.34‐62.49) and 32.25% (95% CI 27.47‐37.64), respectively.

**TABLE 3:** Survival analysis of patients treated with apatinib

Survival	Efficacy (n = 747)
mPFS (95% CI)	5.56 (4.74‐6.28)
6‐mo (%) (95% CI)	47.04 (42.93‐51.05)
12‐mo (%) (95% CI)	22.85 (18.40‐27.60)
mOS (95% CI)	7.5 (6.74‐8.88)
6‐mo (%) (95% CI)	58.54 (54.34‐62.49)
12‐mo (%) (95% CI)	32.25 (27.47‐37.64)

Abbreviations: mOS, median overall survival; mPFS, median progression‐free survival.

An exploratory univariate analysis was carried out by the Kaplan‐Meier analysis and log‐rank test. We found that there were significant association between the mPFS and combined therapy, apatinib suspension, number of metastases sites, and hand‐foot syndrome (all *P* < .05). Also, there were also significant association of mOS with combined therapy, dose adjustment, clinical stage, previous surgery history, hypertension, proteinuria, and hand‐foot syndrome (all *P* < .05). We also observed that apatinib treatment lines were not significantly associated with mPFS and mOS. Detailed results of univariate analysis were shown in Table 4.

**TABLE 4:** Exploratory univariate analysis of factors to predict PFS and OS of apatinib

Variable	Number	mPFS (m)	*P*	mOS (m)	*P*
Total patients	747	5.56		7.5	
Apatinib treatment lines			.61		.80
1	325	5.72		7.63	
2	209	5.52		7.50	
≥3	205	4.87		7.50	
Combined therapy^a^			.01		.02
Yes	338	6.38		8.88	
No	407	4.61		6.51	
Apatinib suspension			.009		.41
No	569	5.95		7.76	
Yes	178	4.18		7.04	
Dose adjustment			.27		.03
Yes	86	7.3		10.63	
No	661	5.33		7.24	
Clinical stage			.15		.04
III	101	6.74		10.43	
IV	489	4.77		7.43	
Previous surgery history			.09		.01
Yes	416	5.69		8.68	
No	331	5.33		7.24	
Number of metastases sites			.01		.98
>2	95	3.45		6.5	
≤2	331	5.69		7.5	
Hypertension			.664		.03
Yes	198	5.26		9.67	
No	322	4.14		7.27	
Proteinuria			.07		.002
Yes	52	7.30		13.62	
No	454	4.28		7.73	
Hand‐foot syndrome			<.001		<.001
Yes	162	8.59		12.99	
No	307	3.09		5.03	

Note

*P* values by log‐rank test are displayed.

Abbreviations: mOS, median overall survival; mPFS, median progression‐free survival.

^a^ Combined with XELOX, 5‐FU, DCF, and EOX regimen.

In addition, as shown in Table 5, multivariate Cox regression analysis indicated that hand‐foot syndrome was one independent predictor for PFS and OS. Also, combination regimen (apatinib plus taxol/docetaxel) was also one independent predictor for PFS.

**TABLE 5:** Multivariate Cox regression analyses for PFS and OS

	PFS			OS		
	*P*	HR	95% CI	*P*	HR	95% CI
Gender	.488	0.844	0.522‐1.363	.954	1.017687	0.561‐1.850
Age	.453	0.860	0.580‐1.275	.137	0.6963783	0.432‐1.122
ECOG score	.833	1.140	0.337‐3.852	.405	1.889048	0.4226‐8.445
Clinical stage	.864	1.051	0.595‐1.855	.558	0.8124894	0.405‐1.629
Surgery history	.989	0.997	0.661‐1.505	.218	0.7334019	0.448‐1.201
Chemotherapy history	.836	1.060	0.610‐1.844	.155	1.63683	0.830‐3.227
Treatment line	.514	0.905	0.672‐1.220	.921	1.019407	0.696‐1.493
Combination regimen	.032*	0.440	0.207‐0.932	.432	0.7238228	0.323‐1.622
Dose adjustment	.124	0.662	0.392‐1.120	.33	0.7285891	0.385‐1.377
Apatinib suspension	.889	1.030	0.681‐1.556	.209	0.727765	0.443‐1.194
Hypertension	.677	0.920	0.619‐1.365	.114	0.6727261	0.411‐1.100
Proteinuria	.204	0.662	0.351‐1.250	.108	0.4579196	0.176‐1.188
Hand‐foot syndrome	0**	0.230	0.139‐0.376	0**	0.1876364	0.102‐0.344
Fatigue	.629	0.892	0.562‐1.416	.454	0.8030185	0.452‐1.427

* *P *< .05

** *P *< .01

### 3.3 Safety

A total of 574 patients were enrolled for the assessment of safety. The occurring frequencies of all adverse events were 73.59%, and the occurring frequencies of grade ≥3 AEs were 18.97%. No unexpected AEs and SAEs were observed. The most common AEs included hypertension (36.55%), proteinuria (10.26%), hand‐foot syndrome (33.53%), fatigue (24.9%), anemia (57.35%), leukopenia (44.49%), thrombocytopenia (34.21%), and neutropenia (53.33%). Grade ≥3 AEs with incidences of more than 5% were anemia (13.97%), thrombocytopenia (7.14%), and neutropenia (6.67%). The detailed AEs were shown in Table 6. In addition, 20 patients were reduced to the 250 mg dose from 500 mg dose and 44 patients were increased to 500 mg dose from 250 mg dose. A total of 178 patients suspended the administration (156 patients suspended one time, 17 suspended two times, and 5 suspended three times).

**TABLE 6:** Adverse events

Adverse events	Any grade(%)	Grade ≥ 3 (%)
Hypertension	36.55	3.82
Fatigue	24.9	2.01
Hand‐foot syndrome	33.53	2.41
Proteinuria	10.26	0.56
Anemia	57.35	13.97
Thrombocytopenia	34.21	7.14
Neutropenia	53.33	6.67
Leukocytopenia	44.49	1.84

## 4 DISCUSSION

Patients with advanced gastric cancer generally showed dismal prognoses. Two‐drug regimens or three‐drug regimens from fluoropyrimidine‐based and platinum‐based chemotherapies have been used as the standard therapy of first line.^9^, ^10^, ^11^ For HER2‐positive advanced gastric or gastroesophageal junction (GEJ) cancer, trastuzumab in combination with chemotherapy dramatically extended the OS of the patients and might be a fresh standard therapy for this disease.^12^ For second‐line therapy for gastric cancer, chemotherapeutic agents such as paclitaxel and irinotecan and antiangiogenic targeted agents such as ramucirumab have been recommended by NCCN guideline.^13^ The second‐line chemotherapy achieved an mOS of 5.8‐9.5 months and an mPFS of 2.2‐3.6 months.^14^ Ramucirumab monotherapy or combination with paclitaxel benefits patients with the mOS of 5.2‐9.6 months.^15^ Apatinib has been approved in patients with chemotherapy‐refractory advanced or metastatic adenocarcinoma of the stomach or GEJ as third‐ or further‐line treatment in China based on the results of a II and III trials, which showed the benefits of mPFS (2.6 months vs 1.8 months, *P*  <  .001) and mOS (6.5 months vs 4.7 months, *P * =  .0149) compared with placebo control.^5^, ^6^

In this real‐world study, our results showed that the mPFS was 5.56 months (95% CI 4.47‐6.28), and mOS was 7.5 months (95% CI 6.74‐8.88), the DCR and ORR was 56.89% and 6.83%, respectively. These data were better than the previous studies. For example, Li et al observed that apatinib achieved an mPFS of 2.6‐3.67 months and mOS of 4.27‐6.5 months in patients with chemotherapy‐refractory advanced or metastatic adenocarcinoma of the stomach or GEJ in a phase II trial and III trial.^5^, ^6^ In addition, in a real‐world study, Zhang et al found that apatinib achieved an mPFS of 2.65 months, an mOS of 5.8 months, and ORR of 5.6% and DCR of 58.3% in 36 patients with advanced gastric adenocarcinoma or adenocarcinoma of GEJ after failing at least two lines of systemic therapy.^16^

In addition, univariate analysis found that mPFS and mOS were significantly associated with combined therapy and hand‐foot syndrome. Combination with chemotherapy included apatinib plus taxol/docetaxel, XELOX, 5‐FU, DCF, and EOX regimen, which would improve the survival of patients.

To exclude the effect of the confounders for survival outcome, we further performed the Cox regression analysis and found that apatinib plus taxol/docetaxel and hand‐foot syndrome were independent significant factors to affect the PFS and hand‐foot syndrome was only one independent significant factor to influence the OS. Li et al reported that apatinib treatment prolonged progression‐free survival and lead to improved OS.^17^ Interestingly, we did not observe the apatinib plus taxol/docetaxel affect the OS by Cox regression analysis. Hand‐foot syndrome has been recognized as a viable biomarker of antitumor efficacy and was associated with prolonged mOS and prolonged mPFS in previous study,^18^ consistently with our data.

In this study, apatinib was prescribed at a low dose of 500 mg or 250mg a day initially, which was lower than the previous reported dose in gastric cancer (850 mg daily or 425 mg twice a day)^5^, ^6^; and a large number of studies have also demonstrated that the dose of apatinib 500 mg per day is effective in other solid tumors, such as thyroid cancer, breast cancer, sarcoma, lung cancer, hepatocellular carcinoma. Thus, this real‐world study further provided the clinical implication for the use of low‐dose apatinib in gastric cancer.

Hypertension, proteinuria, and hand‐foot syndrome are the most common AEs in antiangiogenic therapy.^19^, ^20^, ^21^ The most common AEs in our study are almost similar to those reported in previous studies of apatinib.^5^, ^6^ Besides, hematologic toxicities including anemia (57.35%), leukopenia (44.49%), thrombocytopenia (34.21%), neutropenia (53.33%) also occurred. Grade 3‐4 AEs with incidences of more than 5% were anemia (13.97%), thrombocytopenia (7.14%), and neutropenia (6.67%).

## 5 CONCLUSION

Taken together, the prospective study suggested that low‐dose apatinib was an effective regimen for advanced gastric cancer with manageable toxicity in the real‐world study.

## CONFLICT OF INTEREST

All authors declare no potential conflict of interest.

## AUTHOR CONTRIBUTIONS

All authors were involved in conception and design. All authors were involved in collection and assembly of data. All authors were involved in administrative support, data analysis, and interpretation. All authors approved the final manuscript and accountable for all aspects of the work.

## Data Availability

The datasets used and/or analyzed during the current study are available from the corresponding author on reasonable request. All data generated or analyzed during this study are included in this published article.
